# Umbilical cord-derived Wharton’s jelly for regenerative medicine applications

**DOI:** 10.1186/s13018-020-1553-7

**Published:** 2020-02-13

**Authors:** Ashim Gupta, Saadiq F. El-Amin, Howard J. Levy, Rebecca Sze-Tu, Sobrasua E. Ibim, Nicola Maffulli

**Affiliations:** 1 BioIntegrate, New York, NY USA; 2 South Texas Orthopaedic Research Institute, Laredo, TX USA; 3 Department of Psychology, Illinois Wesleyan University, Bloomington, IL USA; 4 Future Biologics, Lawrenceville, GA USA; 5 El-Amin Orthopaedic and Sports Medicine Institute, Duluth, GA USA; 6 Department of Orthopaedic Surgery, Lenox Hill Hospital, Northwell Health, New York, NY USA; 7 Department of Biomedical Engineering, Columbia University, New York, NY USA; 8 Morris Brown College, Atlanta, GA USA; 9 Department of Musculoskeletal Disorders, School of Medicine and Surgery, University of Salerno, Fisciano, Italy; 10 Queen Mary University of London Barts and the London School of Medicine and Dentistry, Centre for Sports and Exercise Medicine, London, England; 11 Keele University Faculty of Medicine, School of Pharmacy and Bioengineering, Stoke on Trent, England

**Keywords:** Regenerative medicine, Musculoskeletal injuries, Osteoarthritis, Biologics, Umbilical cord, Wharton’s jelly, Growth factors, cytokines, Hyaluronic acid, Exosomes

## Abstract

**Background:**

The last decade has seen an explosion in the interest in using biologics for regenerative medicine applications, including umbilical cord-derived Wharton’s Jelly. There is insufficient literature assessing the amount of growth factors, cytokines, hyaluronic acid, and extracellular vesicles including exosomes in these products. The present study reports the development of a novel Wharton’s jelly formulation and evaluates the presence of growth factors, cytokines, hyaluronic acid, and extracellular vesicles including exosomes.

**Methods:**

Human umbilical cords were obtained from consenting caesarian section donors. The Wharton’s jelly was then isolated from the procured umbilical cord and formulated into an injectable form. Randomly selected samples from different batches were analyzed for sterility testing and to quantify the presence of growth factors, cytokines, hyaluronic acid, and extracellular vesicles.

**Results:**

All samples passed the sterility test. Growth factors including IGFBP 1, 2, 3, 4, and 6, TGF-α, and PDGF-AA were detected. Several immunomodulatory cytokines, such as RANTES, IL-6R, and IL-16, were also detected. Pro-inflammatory cytokines MCSFR, MIP-1a; anti-inflammatory cytokines TNF-RI, TNF-RII, and IL-1RA; and homeostatic cytokines TIMP-1 and TIMP-2 were observed. Cytokines associated with wound healing, ICAM-1, G-CSF, GDF-15, and regenerative properties, GH, were also expressed. High concentrations of hyaluronic acid were observed. Particles in the extracellular vesicle size range were also detected and were enclosed by the membrane, indicative of true extracellular vesicles.

**Conclusion:**

There are numerous growth factors, cytokines, hyaluronic acid, and extracellular vesicles present in the Wharton’s jelly formulation analyzed. The amount of these factors in Wharton’s jelly is higher compared with other biologics and may play a role in reducing inflammation and pain and augment healing of musculoskeletal injuries.

## Background

Ligament, muscle, and tendon injuries produce pain, loss of function, instability, and secondary osteoarthritis [1, 2]. Traditionally, these injuries have been managed using activity modification; physical therapy; pharmacological agents, such as non-steroidal anti-inflammatory drugs, corticosteroids, viscosupplementation, and narcotics; and surgical procedures when conservative management fails [3]. These modalities have limitations and potential side effects [4].

Over the last decade, there has been an increased interest in the use of biologics for regenerative medicine applications [5]. Biologics currently used in clinical practice include platelet-rich plasma, bone marrow aspirate, adipose tissue aspirate, amniotic fluid, amniotic membrane, umbilical cord-derived Wharton’s jelly and cord blood [6, 7]. The healing capabilities of these products are attributed to the presence of stem cells, growth factors, cytokines, hyaluronic acid, and/or extracellular vesicles including exosomes [8].

Stem cells, including mesenchymal stem cells isolated from bone marrow, periosteum, adipose tissue, trabecular bone, and deciduous teeth, have produced marked interest for their applications to regenerative medicine [7]. Stem cells are able to differentiate along specific lineage in response to signal transduction mediated by growth factors and cytokines [8]. Growth factors and cytokines often have overlapping activities. They are able to target mesenchymal, endothelial, and epithelial cells, and can act in an autocrine or paracrine manner [8]. In addition, one cytokine can stimulate the synthesis and release of other cytokines leading to a network of interacting molecules. This complex network of cytokines and growth factors can guide cell division, differentiation, and regeneration of different tissues and organs [8].

Hyaluronic acid, a polysaccharide found in most tissues, is a major component of extracellular matrix of the skin, joints, and eyes [9]. Hyaluronic acid has been used to manage knee osteoarthritis via its chondroprotection, proteoglycan and glycosaminoglycan synthesis, and anti-inflammatory, mechanical, subchondral, and analgesic actions [10].

Exosomes are small extracellular vesicles with diameter ranging from ~ 30 to 150 nm, developed from a sequential process of multivesicular body membrane remodeling [11]. Exosomes are found in multiple body fluids including blood plasma, amniotic fluid, and Wharton’s jelly [12, 13]. Exosomes are secreted from several cell types including stem cells and represent an important mode of intercellular communications [13]. Recently, exosomes have also emerged as an attractive cell-free therapeutic alternative that holds great regenerative potential [14].

The increasing applications of biologic therapies for regenerative medicine have led to considerable marketing, patient demand, and clinical utilization [5]. To be compliant in the United States (U.S.), biologics that adhere to the U.S. Food and Drug Administration (FDA) regulation of human cells, tissues, and cellular and tissue-based products (HCT/Ps) regulated under title 21, part 1271 of the Code of Federal Regulations (CFR), must meet all the criteria under section 361 of Public Health Safety (PHS) Act to be regulated solely under this section [5]. According to this regulation, HCT/Ps must meet the criteria of being minimally manipulated, for homologous use only, not to be combination products, to have no systemic effect, and to be non-dependent on the metabolic activity of the living cells [5]. Despite increased use, there is insufficient literature assessing the amount of growth factors, cytokines, hyaluronic acid and extracellular vesicles including exosomes present in these products, and, more specifically, umbilical cord-derived Wharton’s jelly.

Wharton’s jelly is a primordial mucous connective tissue of the umbilical cord present between the amniotic epithelium and the umbilical vessels [15]. The key role of Wharton’s jelly is to provide cushion, protection, and structural support to umbilical vessels by preventing their compression, torsion, and bending [15]. The umbilical vessels also provide bi-directional flow of oxygen, glucose, and amino acids to developing fetus and aids in depleting the fetus of carbon dioxide and other waste products [15]. This gelatinous substance contains primitive mesenchymal stem cells (MSC) [15] and yields the highest concentration of MSC per milliliter of other allogenic tissues [16]. Wharton’s jelly MSC may be more effective than MSC from adult tissues in the treatment of several conditions, and though safe and efficacious, more studies are required to justify their routine use in the clinics [17]. Wharton’s jelly also contains high amounts of extracellular matrix components, including collagen, hyaluronic acid, and sulfated proteoglycans [18].

The present study reports the results of experiments aimed to characterize a novel umbilical cord-derived Wharton’s jelly formulation and to evaluate the presence of growth factors, cytokines, hyaluronic acid, and extracellular vesicles including exosomes. We hypothesized that numerous growth factors, cytokines, hyaluronic acid, and extracellular vesicles including exosomes are present in Wharton’s jelly; all may play a role in reducing inflammation and pain and augment healing of musculoskeletal injuries.

## Methods

Human umbilical cords were obtained from consenting caesarian section donors following standards established by the FDA and the American Association of Tissue Banks. Donors underwent comprehensive medical, social and blood testing prior to donation. Infectious disease testing was performed at an independent certified laboratory in accordance with the Clinical Laboratory Improvement Amendments of 1988 (CLIA) and 42 CFR part 493 and the FDA. Each donor was tested for HIV I/II Plus O Ab (antibodies to human immunodeficiency virus type 1 & 2), HBsAg (HEPATITIS B surface antigen), HBcAb (hepatitis B core Antibody), HBcTotal, HCV NAT (hepatitis C virus nucleic acid test), HTLV (Human T-lymphotropic virus) I/II Ab, RPR (Rapid plasma reagin) syphilis screening - nontreponemal, CMV (*Cytomegalovirus*), HIV-1/HCV (hepatitis C antibody)/HBV NAT Ultrio, WNV (West Nile Virus) NAT.

The procured umbilical cord was rinsed with saline followed by the removal of blood vessels. The Wharton’s jelly was then isolated from the remaining umbilical cord and formulated into an injectable form using proprietary steps for which patent is pending. All the processing was performed under aseptic conditions. This methodology intends to preserve the structural integrity of Wharton’s jelly and does not include use of digestive enzymes, use of cryoprotectants such as dimethyl sulfoxide (DMSO), or isolation and in vitro expansion of cells. This formulation is prepared according to the criteria of minimal manipulation by FDA, does not include any combination products, and is not intended to depend on the metabolic activity of living cells.

A total of 60 samples from three different batches (20 samples per batch) were tested for sterility at an independent CLIA accredited laboratory, Eurofins VRL Laboratories (Centennial, CO, USA), under United States Pharmacopeia Chapter 71 - Sterility Testing guidelines. Six randomly selected samples from two different batches were sent to an independent laboratory, RayBiotech (Norcross, Georgia, USA), and were analyzed for the presence of growth factors and cytokines using Quantibody® Human Growth Factor Array 1 and Quantibody® Human Inflammation Array 3 respectively. The signals were visualized using Innopsys InnoScan (Carbonne, France) at Cy3 wavelength (~ 550-nm excitation, ~ 570-nm emission). Data were analyzed using Q-Analyzer tool and the concentration of cytokines was determined using serial standard curve provided by the manufacturer (RayBiotech, Norcross, Georgia, USA). In addition, six randomly selected samples from two different batches were analyzed for the presence of hyaluronic acid using Hyaluronan Quantikine ELISA (enzyme-linked immunosorbent assay) kit (R&D systems, Minneapolis, MN, USA) according to the manufacturer’s protocol.

Twelve randomly selected samples from the three different batches were sent to an independent laboratory, Extracellular Vesicle Core at Children’s Hospital Los Angeles (California, USA), and were analyzed by nanoparticle tracking analysis for the presence of particles in the extracellular vesicle size range using Malvern Panalytical Nanosight NS300. These samples were also analyzed after staining with a general fluorescent membrane marker, CellMask Orange™ (Thermo Fisher Scientific, Waltham, MA, USA), as previously described [19].

## Results

All samples passed the sterility test. Growth factors, including, Insulin-like growth factor binding proteins (IGFBP) 1, 2, 3, 4, and 6, transforming growth factor alpha (TGF-α), and platelet-derived growth factor-AA (PDGF-AA) were detected in the formulated Wharton’s jelly (Table 1).

**Table 1:** Growth factors (GFs) expressed in the formulated Wharton’s jelly

Growth factors		Average amount (pg/mL)
IGFBP-3	Insulin-like growth factor binding proteins 3	24,985.5
IGFBP-4	Insulin-like growth factor binding proteins 4	12,302
IGFBP-6	Insulin-like growth factor binding proteins 6	7711.1
IGFBP-2	Insulin-like growth factor binding proteins 2	6900.6
IGFBP-1	Insulin-like growth factor binding proteins 1	5211.4
TGF-α	Transforming growth factor alpha	311.4
HGF	Hepatocyte growth factor	266.6
FGF-7	Fibroblast growth factor	102.2
EG-VEGF	Endocrine gland-derived vascular endothelia growth	32.2
PDGF-AA	Platelet-derived growth factor AA	31.9
VEGF R3	Vascular endothelia growth factor receptor 3	16.8
VEGF	Vascular endothelia growth factor	14.4
β-NGF	Beta nerve growth factor	12.8

The expression of several immunomodulatory cytokines, such as RANTES (regulated on activation, normal T cell expressed and secreted), interleukin 6 receptor (IL-6R), interleukin 16 (IL-16), and interferon gamma (IFN-γ) was also detected (Table 2).

**Table 2:** Immunomodulatory cytokines expressed in the formulated Wharton’s jelly

Immunomodulatory cytokines		Average amount (pg/mL)
RANTES	Regulated upon activation, normally T-expressed, and secreted; aka C-C motif chemokine ligand 5 (CCL5)	551.0
IL-6R	Interleukin 6 receptor	53.3
MIP-1D	Macrophage inflammatory protein 5; aka C-C motif chemokine ligand15 (CCL15)	44.9
SCF R	Stem cell factor, aka KIT Proto-oncogene receptor tyrosine Kinase	40.3
MCSF	Macrophage colony-stimulating factor 1	12.2
IL-16	Interleukin 16	8.7
I-309	C-C motif chemokines ligand1 (CCL1)	3.1
IFN-γ	Interferon gamma	1.8
IL-1β	Interleukin 1 beta	1.3
EOTAXIN	C-C motif chemokine ligand 11, 24, 26 (CC11, 24, 26)	1.3

Additionally, the expression of pro-inflammatory cytokines such as macrophage colony stimulating factor (MCSF), macrophage stimulating protein 1-alpha (MIP-1α); anti-inflammatory cytokines, such as tumor necrosis factor receptor superfamily member 1A and 1B (TNF-RI and TNF-RII), interleukin 1 receptor antagonist (IL-1RA); and homeostatic cytokines, such as tissue inhibitor of metalloproteinase 1 and 2 (TIMP-1 and TIMP-2) was also observed (Table 3).

**Table 3:** Pro-inflammatory, anti-inflammatory and homeostatic cytokines expressed in the formulated Wharton’s jelly

		Average amount (pg/mL)
Pro-inflammatory cytokines		
MCSF	Macrophage colony-stimulating factor	930.8
MIP-1α	Macrophage-stimulating protein 1-alpha; aka C-C motif chemokine ligand 3 (CCL3)	1.2
Anti-inflammatory cytokines		
TNF-RI	Tumor necrosis factor receptor superfamily member 1A	191.6
TNF-RII	Tumor necrosis factor, member 1B	89.8
IL-1RA	Interleukin 1 receptor antagonist	58.8
Homeostatic cytokines		
TIMP-2	Tissue inhibitor of metalloproteinases 2	8663.6
TIMP-1	Tissue inhibitor of metalloproteinases 1	7386.7

Cytokines associated with wound healing including intercellular adhesion molecule 1 (ICAM-1), granulocyte-stimulating factor (G-CSF), growth differentiation factor 15 (GDF-15), and regenerative properties such as growth hormone (GH) were also expressed (Table 4).

**Table 4:** Wound-healing and regenerative cytokines expressed in the formulated Wharton’s jelly

		Average amount (pg/mL)
Wound-healing cytokines		
ICAM-1	Intercellular adhesion molecule-1	1554.9
MCP-1	Monocyte chemotactic protein-1, aka CC motif chemokine ligand 2 (CCL2 Gene)	119.0
G-CSF	Granulocyte-stimulating factor, aka Colony-stimulating factor 3 (CSF3)	91.6
GDF-15	Growth differentiation factor 15	89.2
NT-4	Neurotropin-4	33
Regenerative cytokines		
GH	Growth hormone or somatotropin, aka human growth hormone (hGH or HGH)	31.1
GDNF	Glia cell-derived neurotrophic factor	19.5

**Fig. 1:**
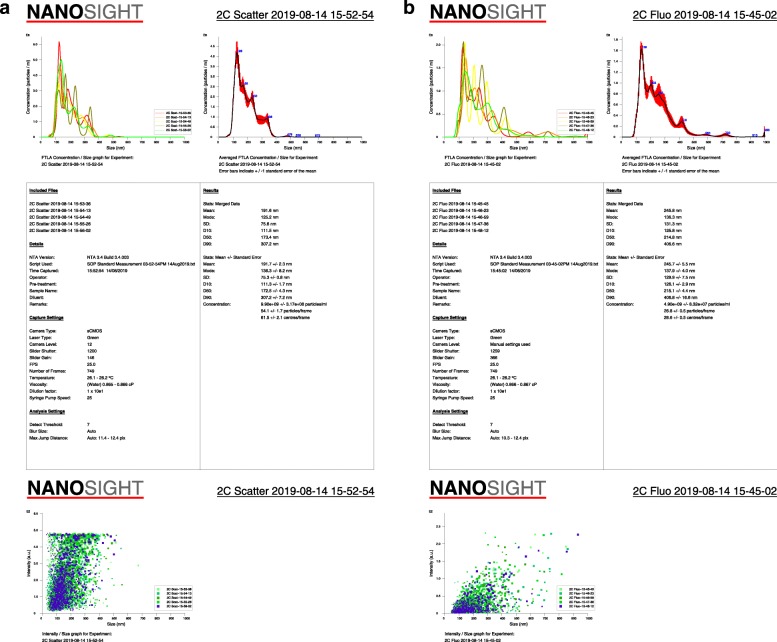
**a** A representative nanoparticle tracking analysis showed the presence of 9.90 ± 0.32 billion particles/mL in the light scattering mode with a mode size of 136.3 ± 8.2 nm. **b** A representative nanoparticle tracking analysis showed the presence of 4.90 ± 0.08 billion particles/mL in the fluorescent mode with a mode size of 137.9 ± 4.0 nm. Values are shown as mean ± standard error

Hyaluronic acid (average amount of 8.7 μg/mL) was detected in the formulated Wharton’s jelly. The nanoparticle tracking analysis demonstrated the presence of billions of particles (average amount of 17.4 billion/mL) in the extracellular vesicles size range in the light scattering mode. CellMask Orange™ staining showed the presence of 4.18 billion particles/mL in the fluorescent mode, indicative of true membrane-enclosed particles, i.e. extracellular vesicles. Representative images for nanoparticle tracking analysis in the light scattering and fluorescent mode are shown as Fig. 1a and b, respectively.

## Discussion

Biologics hold great potential in treating a variety of musculoskeletal ailments [6]. At present, the published literature related to umbilical cord-derived Wharton’s jelly focuses on the isolated cells, and despite the commercial use, there is still insufficient characterization of these formulations [18, 20, 21]. In the present study, we formulated a novel umbilical cord-derived Wharton’s jelly product and evaluated it for the presence of growth factors, cytokines, hyaluronic acid and extracellular vesicles including exosomes. The essential components of regenerative medicine, namely growth factors, cytokines, hyaluronic acid and extracellular vesicles, are all present in the formulated Wharton’s jelly. The results from this study are an essential preliminary first step to better characterize Wharton’s jelly. This is necessary to perform clinical trials to determine safety and efficacy of this novel formulation for regenerative medicine applications.

Numerous growth factors were detected in our Wharton’s jelly formulation. We detected IGFBP 1, 2, 3, 4 and 6, which acts as a carrier protein for insulin-like growth factor – 1 (IGF-1). IGF-1 improves osteogenic differentiation, induces chondrogenic differentiation of mesenchymal stem cells, and stimulates extracellular matrix production [22]. We also detected TGF-α, a transforming growth factor which is a ligand for the epidermal growth factor receptor (EGFR). EGFR promotes proliferation and survival of osteoprogenitors and plays an anabolic role in bone metabolism [23]. In addition, platelet-derived growth factor-AA (PDGF-AA), a potent mitogen for cells of mesenchymal origin, was detected. PDGF-AA exhibits chemotactic effects toward human osteoblasts, and its downregulation is associated with cartilage degeneration [24]. We also detected expression of vascular endothelial growth factor (VEGF), a signal protein produced by cells to stimulate blood vessel formation. VEGF is involved in bone tissue remodeling and new bone formation and is downregulated in patients with osteoarthritis [25].

Several immunomodulatory cytokines essential for regenerative medicine were identified. We detected high levels of chemokine (C-C motif) ligand 5 (CCL5), also known as RANTES (regulated on activation, normal T cell expressed and secreted), which has been reported to be involved in modulation of macrophage phenotype from M1 (pro-inflammatory) to M2 (tissue healing) leading to enhanced osteogenesis [26]. RANTES also plays a vital role in chemotaxis, survival of osteoblasts and bone remodeling [27]. We also observed expression of interleukin 6 receptor (IL-6R). IL-6 plays an important role in immune regulation and tissue regeneration, and, when binding with IL-6R, it activates the downstream STAT3 signaling pathway that promotes osteogenic differentiation in mesenchymal stem cells via autocrine/paracrine feedback loop [28].

We detected pro-inflammatory and anti-inflammatory cytokines in the formulated Wharton’s jelly. Pro-inflammatory cytokines usually exert deleterious effects, including mediation of foreign body response and initiating inflammatory response against implants leading to their premature failure. Recent studies have explored their potential as initiators of regeneration. These studies have proposed a pro-regenerative function of the inflammatory signals initiated by these cytokines, and that a proper sequence of inflammatory signals followed by anti-inflammatory signals is essential for proper healing [29]. We detected macrophage colony-stimulating factor (MCSF), as well as macrophage stimulating protein 1-alpha (MIP1-α), which are essential for osteoclast formation [30, 31]. Osteoclasts play a vital role during early bone healing: they maintain and improve the structural strength of bone tissue in conjunction with osteoblasts in a fine adjusted system [32].

We also identified interleukin 1 receptor antagonist (IL-1RA), a specific interleukin-1 (IL-1) receptor antagonist that competitively binds to the same receptor as IL-1 (including inflammatory IL-α and IL-1β), thereby blocking IL-1 mediated cellular changes [33]. IL-1RA attenuates or prevents cytokine-mediated inflammatory hyperalgesia [34]. Intraarticular injection of IL-1RA in patients with knee osteoarthritis slow its progression while improving pain and WOMAC (The Western Ontario and McMaster Universities Osteoarthritis Index) global score [35]. We detected homeostatic cytokines, tissue inhibitor of metalloproteinases (TIMP) 1 and 2, which regulate the activity of matrix metalloproteinases (MMP) [36]. MMPs can degrade all components of connective tissue at physiological pH and may be involved in bone matrix degradation [37]. TIMPs are downregulated in aged tendons, and mechanical stresses, including injuries, further reduce their levels [38]. In addition, TIMPs regulate several biological processes such as cell growth, differentiation and apoptosis that are independent of its MMP activity [39].

We identified several cytokines involved in wound healing. For example, we detected intercellular adhesion molecule-1 (ICAM-1), which promotes leukocyte accumulation into the wound site required for wound healing [40]. ICAM-1 also has immunosuppressive effects on dendritic cells and T cells, which may aid in the treatment of graft versus host diseases [41]. We detected expression of monocyte chemotactic protein-1 (aka CCL2), a pro-inflammatory cytokine, which promotes wound healing, including in hard to heal diabetic wounds [42]. We detected growth differentiation factor 15 (GDF-15), one of the members of transforming growth factor beta superfamily, which modulates bone microenvironment, including suppression of formation or activation of osteoclasts leading to accumulation of bone matrix [43]. We also detected regenerative cytokines, including growth hormone, which stimulates cell growth, reproduction and regeneration, and plays an important role in cartilage regeneration [44].

We detected expression of hyaluronic acid. The umbilical cord tissue contains high molecular weight hyaluronic acid (HMW), which is associated with high fluid retention in joints and has strong anti-inflammatory properties [45]. In addition, it is useful in the management of knee osteoarthritis via its chondroprotection, proteoglycan and glycosaminoglycan synthesis, and anti-inflammatory, mechanical, subchondral, and analgesic actions [10]. Hyaluronic acid accelerates tendon-to-bone healing after rotator cuff repair and has shown potential in the treatment of enthesopathies such as lateral epicondylitis, patellar tendinopathy, insertional Achilles tendinopathy and plantar fasciitis [46].

We also detected the presence of membrane-enclosed particles in the extracellular vesicle size range. Extracellular vesicles including exosomes have demonstrated potential anti-inflammatory and pro-regenerative effects essential for inducing healing in different tissue types [47]. They positively affect cell proliferation and viability, angiogenesis, and immunomodulation in different physiological systems [47]. Exosome uptake by cells significantly reduces pro-inflammatory gene expression and level of M1 phenotypic marker, increase cell migration, and increase expression of osteogenic markers, which play a unique osteo-immunomodulatory role in regulation of bone dynamics [48]. Exosomes stimulate secretion of favorable cellular factors required to accelerate the healing response for tendon injuries including rotator cuff tears [49, 50]. Exosomes also promote cartilage repair and chondrocyte proliferation in osteoarthritis [51].

These results confirmed our hypothesis that growth factors, cytokines, hyaluronic acid, and extracellular vesicles are present in the formulated Wharton’s jelly. Several published basic science and preliminary clinical studies indicate that the combination of these factors may have added advantages for regenerative medicine applications [46]. For example, a co-injection of growth hormone and hyaluronic acid was more effective in treating osteoarthritis compared with injections of hyaluronic acid alone [52], demonstrating the advantage of different factors in one formulation.

We also compared the amount of growth factors, cytokines, hyaluronic acid, and exosomes in Wharton’s jelly with other biologics based on the published literature. The amount of growth factors in Wharton’s jelly is higher compared with the umbilical cord artery [18]. Jin et al. demonstrated biological advantages of umbilical cord-derived tissue compared with bone marrow- and adipose-derived tissue [53]. Wharton’s jelly-derived tissue offers many advantages over bone marrow-derived tissue [54]. This is attributed to upregulation of genes involved in wound healing and immune response in Wharton’s jelly compared with bone marrow-derived tissue [54]. Amable et al. demonstrated higher expression of factors including RANTES, MCP-1, IL-1RA, and PDGF-AA in supernatant derived from Wharton’s jelly stromal cells compared with bone marrow- and adipose-derived stromal cell supernatant [55]. The amount of VEGF, MCSF, RANTES and MCP-1 is higher in our formulation compared with the amount reported by Amable et al. in the activated platelet-rich plasma (PRP) in another study [56]. Cryopreserved amniotic membrane secreted intermediate levels of TIMP1 and TIMP2, low levels of MCP-1, and no detectable levels of RANTES [57]. In contrast, our formulation expressed high levels of these growth factors and cytokines. The amount of hyaluronic acid detected in our formulation is much higher compared with the amount found in amniotic fluid [58]. Other biologics, such as PRP and bone marrow aspirate concentrate, lack hyaluronic acid. Combining these biologics with hyaluronic acid can further improve the functional outcomes in the management of knee osteoarthritis [59]. The total exosome yield adjusted to 1 million mesenchymal stem cells was 1.3 times higher in amniotic fluid compared with bone marrow [60]. The amount of exosome particles/mL released by 1 million amniotic fluid stem cells is estimated to be 0.3 billion [61]. In contrast, the amount of exosome particles/mL adjusted to 1 million Wharton’s jelly mesenchymal stem cells is around 4 billion [62], higher than both amniotic fluid- and bone marrow-derived stem cells. The amount of growth factors, cytokines, hyaluronic acid, and exosomes in Wharton’s jelly are therefore higher compared with other biologics.

Our study has several limitations. Basic science studies have demonstrated the presence of a large number of growth factors in Wharton’s jelly [18]. However, the assay kits used in our analysis can detect only 40 growth factors and 40 cytokines. Future studies are required to determine other growth factors and cytokines expressed in this formulation. Another limitation is the possible presence of microvesicles in addition to the exosomes in the detected extracellular vesicles. Further analysis is needed to confirm the presence of exosomes using exosome-specific markers via immunoblotting assay. In addition to hyaluronic acid, the extracellular matrix of Wharton’s jelly contains a significant amount of collagen and sulphated glycosaminoglycans required for regenerative medicine applications [20, 21]. Future studies are required to determine the amount of these extracellular matrix components in our formulation and examine their benefits.

## Conclusion

Our Wharton’s jelly formulation demonstrated the presence of growth factors, cytokines, hyaluronic acid, and extracellular vesicles in clinically relevant quantities, in amounts greater compared with other biologics. The presence of multiple factors within one formulation may help reduce inflammation, decrease pain and augment healing of musculoskeletal injuries. These factors represent potential expanded applications for regenerative medicine.

## Abbreviations

CFR: Code of Federal Regulations
CLIA: Clinical Laboratory Improvement Amendments
CMV: *Cytomegalovirus*
DMSO: Dimethyl sulfoxide
EGFR: Epidermal growth factor receptor
ELISA: Enzyme-linked immunosorbent assay
FDA: Food and Drug Administration
G-CSF: Granulocyte-stimulating factor
GDF-15: Growth differentiation factor 15
GH: Growth hormone
HBcAb: Hepatitis B core antibody
HBsAg: Hepatitis B surface antigen
HCT/P’s: Human cells, tissues, and cellular and tissue-based products
HCV NAT: Hepatitis C virus nucleic acid test
HIV I/II Plus O Ab: Antibodies to human immunodeficiency virus type 1 & 2
HMW: High molecular weight
HTLV: Human T-lymphotropic virus
ICAM: Intercellular adhesion molecule
IFN-γ: Interferon gamma
IGF-1: Insulin-like growth factor 1
IGFBP: Insulin-like growth factor binding proteins
IL-16: Interleukin 16
IL-1RA: Interleukin 1 receptor antagonist
IL-6R: Interleukin 6 receptor
MCP-1: Monocyte chemotactic protein 1
MCSF: Macrophage colony-stimulating factor
MIP-1α: Macrophage stimulating protein 1-alpha
MMP: Matrix metalloproteinases
PDGF-AA: Platelet-derived growth factor-AA
PHS: Public Health Safety
PRP: Platelet-rich plasma
RANTES: Regulated on activation, normal T cell expressed and secreted
RPR: Rapid plasma reagin
TGF-α: Transforming growth factor
TIMP: Tissue inhibitor of metalloproteinase 1 and 2
TNF: Tumor necrosis factor
U.S.: United States
VEGF: Vascular endothelial growth factor
WNV: West Nile Virus
WOMAC: Western Ontario and McMaster Universities Osteoarthritis Index

## References

[1] Gupta A, Sharif K, Walters M, Woods MD, Potty A, Main BJ, et al. Surgical Retrieval, Isolation and In vitro Expansion of Human Anterior Cruciate Ligament-derived Cells for Tissue Engineering Applications. J Vis Exp. 2014;(86). 10.3791/51597. 10.3791/51597 PMC4184405 24836540

[2] Maffulli N, Oliva F, Frizziero A, Nanni G, Barazzuol M, Via AG (2014). ISMuLT guidelines for muscle injuries. Muscles Ligaments Tendons J.

[3] Baoge L, Van Den Steen E, Rimbaut S, Philips N, Witvrouw E, Almqvist KF (2012). Treatment of skeletal muscle injury: a review. ISRN Orthop.

[4] Eyichukwu GO (2010). Non-steroidal anti inflammatory drugs usage in orthopaedics and trauma practice. A guide and review. Niger J Med.

[5] Lamplot Joseph D., Rodeo Scott A., Brophy Robert H. (2019). A Practical Guide for the Current Use of Biologic Therapies in Sports Medicine. The American Journal of Sports Medicine.

[6] Sezgin EA, Atik OŞ (2018). Are orthobiologics the next chapter in clinical orthopedics? A literature review. Eklem Hastalik Cerrahisi.

[7] Chirichella PS, Jow S, Iacono S, Wey HE, Malanga GA (2019). Treatment of knee meniscus pathology: rehabilitation, surgery, and orthobiologics. PM&R..

[8] Ioannidou E (2006). Therapeutic modulation of growth factors and cytokines in regenerative medicine. Curr Pharm Des.

[9] Fraser JR, Laurent TC, Laurent UB (1997). Hyaluronan: its nature, distribution, functions and turnover. J Intern Med.

[10] Altman RD, Manjoo A, Fierlinger A, Niazi F, Nicholls M (2015). The mechanism of action for hyaluronic acid treatment in the osteoarthritic knee: a systematic review. BMC Musculoskelet Disord.

[11] Bittel DC, Jaiswal JK (2019). Contribution of extracellular vesicles in rebuilding injured muscles. Front Physiol.

[12] Caby M-P, Lankar D, Vincendeau-Scherrer C, Raposo G, Bonnerot C (2005). Exosomal-like vesicles are present in human blood plasma. Int Immunol.

[13] Raposo G, Stoorvogel W (2013). Extracellular vesicles: exosomes, microvesicles, and friends. J Cell Biol.

[14] Matei AC, Antounians L, Zani A. Extracellular vesicles as a potential therapy for neonatal conditions: state of the art and challenges in clinical translation. Pharmaceutics. 2019;11(8). 10.3390/pharmaceutics11080404. 10.3390/pharmaceutics11080404 PMC6723449 31405234

[15] Taghizadeh RR, Cetrulo KJ, Cetrulo CL (2011). Wharton’s jelly stem cells: future clinical applications. Placenta.

[16] Vangsness CT, Sternberg H, Harris L (2015). Umbilical cord tissue offers the greatest number of harvestable Mesenchymal stem cells for research and clinical application: a literature review of different harvest sites. Arthroscopy.

[17] Liau L.L., Ruszymah B.H.I., Ng M.H., Law J.X. (2020). Characteristics and clinical applications of Wharton’s jelly-derived mesenchymal stromal cells. Current Research in Translational Medicine.

[18] Sobolewski K, Małkowski A, Bańkowski E, Jaworski S (2005). Wharton’s jelly as a reservoir of peptide growth factors. Placenta..

[19] Carnell-Morris P, Tannetta D, Siupa A, Hole P, Dragovic R (1660). Analysis of extracellular vesicles using fluorescence nanoparticle tracking analysis. Methods Mol Biol.

[20] Sobolewski K, Bańkowski E, Chyczewski L, Jaworski S (1997). Collagen and glycosaminoglycans of Wharton’s jelly. Biol Neonate.

[21] Gogiel T, Bańkowski E, Jaworski S (2003). Proteoglycans of Wharton’s jelly. Int J Biochem Cell Biol.

[22] Fortier LA, Barker JU, Strauss EJ, McCarrel TM, Cole BJ (2011). The role of growth factors in cartilage repair. Clin Orthop Relat Res.

[23] Zhang X, Tamasi J, Lu X, Zhu J, Chen H, Tian X (2011). Epidermal growth factor receptor plays an anabolic role in bone metabolism in vivo. J Bone Miner Res.

[24] Lind M (1998). Growth factor stimulation of bone healing. Effects on osteoblasts, osteomies, and implants fixation. Acta Orthop Scand Suppl.

[25] Wyatt LA, Nwosu LN, Wilson D, Hill R, Spendlove I, Bennett AJ (2019). Molecular expression patterns in the synovium and their association with advanced symptomatic knee osteoarthritis. Osteoarthr Cartil.

[26] Córdova LA, Loi F, Lin T-H, Gibon E, Pajarinen J, Nabeshima A (2017). CCL2, CCL5, and IGF-1 participate in the immunomodulation of osteogenesis during M1/M2 transition in vitro. J Biomed Mater Res A.

[27] Liu Y-C, Kao Y-T, Huang W-K, Lin K-Y, Wu S-C, Hsu S-C (2014). CCL5/RANTES is important for inducing osteogenesis of human mesenchymal stem cells and is regulated by dexamethasone. Biosci Trends.

[28] Rincon M (2012). Interleukin-6: from an inflammatory marker to a target for inflammatory diseases. Trends Immunol.

[29] Spiller KL, Nassiri S, Witherel CE, Anfang RR, Ng J, Nakazawa KR (2015). Sequential delivery of immunomodulatory cytokines to facilitate the M1-to-M2 transition of macrophages and enhance vascularization of bone scaffolds. Biomaterials..

[30] Fujikawa Y, Sabokbar A, Neale SD, Itonaga I, Torisu T, Athanasou NA (2001). The effect of macrophage-colony stimulating factor and other humoral factors (interleukin-1, -3, -6, and -11, tumor necrosis factor-alpha, and granulocyte macrophage-colony stimulating factor) on human osteoclast formation from circulating cells. Bone..

[31] Dapunt U, Maurer S, Giese T, Gaida MM, Hänsch GM (2014). The macrophage inflammatory proteins MIP1α (CCL3) and MIP2α (CXCL2) in implant-associated osteomyelitis: linking inflammation to bone degradation. Mediat Inflamm.

[32] Schell H, Lienau J, Epari DR, Seebeck P, Exner C, Muchow S (2006). Osteoclastic activity begins early and increases over the course of bone healing. Bone..

[33] Zhang J-M, An J (2007). Cytokines, inflammation, and pain. Int Anesthesiol Clin.

[34] Maier SF, Wiertelak EP, Martin D, Watkins LR (1993). Interleukin-1 mediates the behavioral hyperalgesia produced by lithium chloride and endotoxin. Brain Res.

[35] Frizziero A, Giannotti E, Oliva F, Masiero S, Maffulli N (2013). Autologous conditioned serum for the treatment of osteoarthritis and other possible applications in musculoskeletal disorders. Br Med Bull.

[36] Bord S, Horner A, Hembry RM, Reynolds JJ, Compston JE (1997). Distribution of matrix metalloproteinases and their inhibitor, TIMP-1, in developing human osteophytic bone. J Anat.

[37] Everts V, Delaissé JM, Korper W, Niehof A, Vaes G, Beertsen W (1992). Degradation of collagen in the bone-resorbing compartment underlying the osteoclast involves both cysteine-proteinases and matrix metalloproteinases. J Cell Physiol.

[38] Ueda Y, Inui A, Mifune Y, Takase F, Kataoka T, Kurosawa T (2019). Molecular changes to tendons after collagenase-induced acute tendon injury in a senescence-accelerated mouse model. BMC Musculoskelet Disord.

[39] Stetler-Stevenson WG (2008). Tissue inhibitors of metalloproteinases in cell signaling: metalloproteinase-independent biological activities. Sci Signal.

[40] Nagaoka T, Kaburagi Y, Hamaguchi Y, Hasegawa M, Takehara K, Steeber DA (2000). Delayed wound healing in the absence of intercellular adhesion molecule-1 or L-selectin expression. Am J Pathol.

[41] Tang B, Li X, Liu Y, Chen X, Li X, Chu Y (2018). The therapeutic effect of ICAM-1-overexpressing mesenchymal stem cells on acute graft-versus-host disease. Cell Physiol Biochem.

[42] Wood S, Jayaraman V, Huelsmann EJ, Bonish B, Burgad D, Sivaramakrishnan G (2014). Pro-inflammatory chemokine CCL2 (MCP-1) promotes healing in diabetic wounds by restoring the macrophage response. PLoS One.

[43] Vanhara P, Lincová E, Kozubík A, Jurdic P, Soucek K, Smarda J (2009). Growth/differentiation factor-15 inhibits differentiation into osteoclasts--a novel factor involved in control of osteoclast differentiation. Differentiation.

[44] Lubis AMT, Wonggokusuma E, Marsetio AF (2019). Intra-articular recombinant human growth hormone injection compared with hyaluronic acid and placebo for an osteoarthritis model of New Zealand rabbits. Knee Surg Relat Res.

[45] Gigis I, Fotiadis E, Nenopoulos A, Tsitas K, Hatzokos I (2016). Comparison of two different molecular weight intra-articular injections of hyaluronic acid for the treatment of knee osteoarthritis. Hippokratia..

[46] Frizziero A, Vittadini F, Oliva F, Abatangelo G, Bacciu S, Berardi A (2018). I.S.Mu.L.T. Hyaluronic acid injections in musculoskeletal disorders guidelines. MLTJ.

[47] Li Jiao, Hosseini-Beheshti Elham, Grau Georges, Zreiqat Hala, Little Christopher (2019). Stem Cell-Derived Extracellular Vesicles for Treating Joint Injury and Osteoarthritis. Nanomaterials.

[48] Wei F, Li Z, Crawford R, Xiao Y, Zhou Y (2019). Immunoregulatory role of exosomes derived from differentiating mesenchymal stromal cells on inflammation and osteogenesis. J Tissue Eng Regen Med.

[49] Connor DE, Paulus JA, Dabestani PJ, Thankam FK, Dilisio MF, Gross RM (2019). Therapeutic potential of exosomes in rotator cuff tendon healing. J Bone Miner Metab.

[50] Tetta C, Consiglio AL, Bruno S, Tetta E, Gatti E, Dobreva M (2012). The role of microvesicles derived from mesenchymal stem cells in tissue regeneration; a dream for tendon repair?. Muscles Ligaments Tendons J.

[51] Liu Y, Zou R, Wang Z, Wen C, Zhang F, Lin F (2018). Exosomal KLF3-AS1 from hMSCs promoted cartilage repair and chondrocyte proliferation in osteoarthritis. Biochem J.

[52] Kim SB, Kwon DR, Kwak H, Shin YB, Han H-J, Lee JH (2010). Additive effects of intra-articular injection of growth hormone and hyaluronic acid in rabbit model of collagenase-induced osteoarthritis. J Korean Med Sci.

[53] Jin HJ, Bae YK, Kim M, Kwon S-J, Jeon HB, Choi SJ (2013). Comparative analysis of human mesenchymal stem cells from bone marrow, adipose tissue, and umbilical cord blood as sources of cell therapy. Int J Mol Sci.

[54] Barrett AN, Fong C-Y, Subramanian A, Liu W, Feng Y, Choolani M (2019). Human Wharton’s jelly Mesenchymal stem cells show unique gene expression compared with bone marrow Mesenchymal stem cells using single-cell RNA-sequencing. Stem Cells Dev.

[55] Amable PR, Teixeira MVT, Carias RBV, Granjeiro JM, Borojevic R (2014). Protein synthesis and secretion in human mesenchymal cells derived from bone marrow, adipose tissue and Wharton’s jelly. Stem Cell Res Ther.

[56] Amable PR, Carias RBV, Teixeira MVT, da Cruz PI, do Amaral RJF C, Granjeiro JM (2013). Platelet-rich plasma preparation for regenerative medicine: optimization and quantification of cytokines and growth factors. Stem Cell Res Ther.

[57] Wolbank S, Hildner F, Redl H, van Griensven M, Gabriel C, Hennerbichler S (2009). Impact of human amniotic membrane preparation on release of angiogenic factors. J Tissue Eng Regen Med.

[58] Pierce J, Jacobson P, Benedetti E, Peterson E, Phibbs J, Preslar A (2016). Collection and characterization of amniotic fluid from scheduled C-section deliveries. Cell Tissue Bank.

[59] Papalia R, Zampogna B, Russo F, Torre G, De Salvatore S, XIX CONGRESSO NAZIONALE S.I.C.O.O.P. SOCIETA’ ITALIANA CHIRURGHI ORTOPEDICI DELL’OSPEDALITA’ PRIVATA ACCREDITATA (2019). The combined use of platelet rich plasma and hyaluronic acid: prospective results for the treatment of knee osteoarthritis. J Biol Regul Homeost Agents.

[60] Tracy SA, Ahmed A, Tigges JC, Ericsson M, Pal AK, Zurakowski D (2019). A comparison of clinically relevant sources of mesenchymal stem cell-derived exosomes: bone marrow and amniotic fluid. J Pediatr Surg.

[61] Balbi C, Piccoli M, Barile L, Papait A, Armirotti A, Principi E (2017). First characterization of human amniotic fluid stem cell extracellular vesicles as a powerful paracrine tool endowed with regenerative potential. Stem Cells Transl Med.

[62] Bellio MA, Sharma M, Lee Y, Kulandavelu S, Benny M, Young K (2019). Bioreactor produced Wharton’s jelly mesenchymal stem cell- derived exosomes prevent lung injury in a pre-clinical model of bronchopulmonary dysplasia. Cytotherapy..

